# Resveratrol fails to provide prophylactic protection in a rat model of organophosphate poisoning

**DOI:** 10.1186/s40696-016-0021-8

**Published:** 2016-07-26

**Authors:** Yossi Rosman, Shaul Ravfogel, Arthur Shiyovich, Shai Shrot, Nadav Milk, Nimrod Ophir, Michael Aviram, Ishai Nir, Michael Kassirer, Arik Eisenkraft

**Affiliations:** 1 Israel Defense Forces Medical Corps, Tel Hashomer, Israel; 2 The Sackler Faculty of Medicine, Tel Aviv University, Tel Aviv, Israel; 3 Lipid Research Laboratory, Technion Faculty of Medicine, Rambam Medical Center, Bat-Galim, Haifa, Israel; 4 The Institute for Research in Military Medicine, The Faculty of Medicine, The Hebrew University of Jerusalem, Jerusalem, Israel; 5 NBC Protection Division, Israel Ministry of Defense, Kaplan St., Hakirya, Tel Aviv, 61909 Israel

**Keywords:** Organophosphates, Nerve agents, Pre-treatment, Prophylaxis, Bioscavengers, Medical countermeasure, Resveratrol, PON-1

## Abstract

**Background:**

Paraoxonase-1, an organophosphorous-hydrolyzing enzyme, was shown to provide protection against organophosphates poisoning in vivo. In vitro findings suggest that the phytoalexin resveratrol can elevate paraoxonase-1 levels and thus may provide protection against organophosphate poisoning. This study was conducted to evaluate the effect of prolonged resveratrol intake on paraoxonase-1 levels in rats, and its role as a potential prophylactic treatment in organophosphate poisoning.

**Methods:**

30 adult male albino Sprague–Dawley rats were randomly assigned into three groups: rats receiving no resveratrol (Control group, n = 10), rats treated once daily with oral gavage of ethanol only (Sham group, n = 6), and rats treated once daily with oral gavage of resveratrol (50 mg/kg) (Study group, n = 14). Following 2 weeks of feeding, all rats were exposed to 1.4LD50 paraoxon (450 mg/kg, intramuscular; 0.5 ml/kg) and monitored for severity of clinical signs and mortality. Paraoxonase-1 activity level was recorded in the beginning of the study and 2 weeks later, just before exposure to paraoxon.

**Results:**

We found a significant decrease in paraoxonase-1 activity levels in all groups compared to baseline levels (p = 0.05), but no significant difference was observed between the study group and the controls (p = 0.7). Following exposure to paraoxon, all animals suffered from severe convulsions and died within minutes.

**Conclusions:**

Following resveratrol intake in rats, paraoxonase-1 activity levels decreased. We found no beneficial effects in using resveratrol as a prophylactic medical countermeasure.

## Background

Organophosphate (OP) nerve agents (NAs) pose an imminent threat to both warfighters and civilian population [1]. Due to the toxic nature of NAs, efforts are aimed at introducing advanced pre-treatment and prophylactic medical countermeasures to the field, which will enable enhanced survival with less morbidity of soldiers in case of exposure. Pyridostigmine bromide is an accepted pre-treatment measure [2]. However, it provides only minor protection against OP poisoning, and helps mainly through enhancement of the protection provided by the post exposure antidotal mixture [2]. It does not protect from OP-induced major CNS symptoms and is not considered to be a prophylactic measure [3]. These drawbacks have resulted in active search of substitute compounds.

An ideal prophylactic compound will enable sufficient levels of functional cholinesterase; is expected to be active against a wide range of OPs and NAs; provide protection by itself, even without any post-exposure treatment; can be taken in a simple way; have a high safety profile and without side-effects; and will provide a long-lasting effect [4–6].

There are several families of prophylactic compounds, defined based on their mode of action. Detoxifying prophylactic compounds include stoichiometric and catalytic bioscavengers. Stoichiometric scavengers bind permanently and neutralize the OPs. These include acetylcholine esterase (AChE) and butyrylcholine esterase (BChE), with effects against all OPs, including NAs [7]. Several transgenic and recombinant systems were developed so far [8, 9] but none have reached full maturity and marketing due to several draw-backs, mainly in upscale techniques, immunogenicity, shelf life, large quantities needed for effective treatment, and price. Catalytic scavengers include several hydrolytic enzymes [10]. Unlike the stoichiometric scavengers, a relatively low dose of the catalytic scavengers should suffice to hydrolyze large quantities of OPs [11]. Despite good results in terms of preventing clinical poisoning [12, 13], these compounds are less broad-spectrum in nature, though efforts are made to make them as such [14, 15]. One of the candidate catalytic scavengers is human paraoxonase-1 (PON-1), a high-density lipoprotein-associated calcium-dependent organophosphate-hydrolase [16], which is mainly induced and secreted by the liver [17]. It was shown already to have a potential protective effect against OP poisoning [18]. Moreover, lack of PON-1 in mice increased OP toxicity [19]. The induction of this endogenous enzyme biosynthesis, has led to increased activity of the human PON-1 gene promoter, followed by elevated activity of serum PON-1, providing protection against OP exposure in rats [18].

The phytoalexin resveratrol is a potent antioxidant that displays antiplatelet, anti-neoplastic, anti-inflammatory and neuroprotective properties [20, 21], and is commercially available as a food supplement. Gouedard et al. showed increased in vitro expression of the HuPON-1 gene following exposure to resveratrol [22]. Moreover, increased expression of human PON-1 resulted in in vitro protection against soman and sarin simulants [23]. To The best of our knowledge there are no published data regarding resveratrol’s ability to increase plasma levels of PON-1 in vivo.

The aim of the current preliminary study was to assess whether pre-treatment with resveratrol can provide protection against OP exposure in a rat model.

## Methods

### Animals

Following institutional review board approval, 30 adult male albino Sprague–Dawley rats (300–320 g, Harlan-biotech, Jerusalem, Israel) were housed under standard laboratory conditions in plastic cages, three per cage, in a controlled environment with a constant temperature of 21 ± 2 °C and 12 h light/dark cycles. Food (Teklad certified global 18 % protein) and water were available ad libitum. All experiments were designed and performed in accordance with the statutes from “The guide for the care and use of laboratory animals in research” of the Ben-Gurion University of the Negev. This study complies with the Animal Welfare Act and other federal regulations which were approved by the Animal Care Committee. All procedures were performed under the supervision of a veterinarian.

### Drugs

Paraoxon was purchased from Sigma Chemical Co. (Israel) and diluted in a vehicle containing 40 % propylene glycol. Resveratrol was purchased in a commercially-available form of capsules, each containing 100 mg resveratrol dissolved in ethanol (Solgar, Israel).

### Study design

Animals were randomly divided into three groups: (1) rats receiving no resveratrol (Control group, n = 10); (2) rats treated once daily with oral gavage of ethanol only (Sham group, n = 6); and (3) rats treated once daily with oral gavage of resveratrol (50 mg/kg) (Study group, n = 14). Following 2 weeks of feeding, during which animals’ weight was followed, all rats were exposed to 1.4LD50 paraoxon (450 mg/kg, intramuscular; 0.5 ml/kg) and monitored for severity of clinical signs and mortality. Clinical score was defined as follows: (0) no activity, quiet periods; (1) chewing and facial clonus; (2) tremors and focal convulsions; and (3) severe generalized tonic–clonic convulsions.

### Laboratory tests

PON-1 activity level was measured in the beginning of the study and 2 weeks after the oral supplementation of resveratrol, just before exposure to paraoxon. PON-1 activity level was analyzed as described by Aviram and Rosenblat [24]. Briefly, 5 µl aliquots of serum were analyzed. The basal assay mixture included 1.0 mM phenyl acetate in “activity buffer”. Phenyl acetate hydrolysis was kinetically monitored for 3 min (every 15 s) at 270 nm. One unit of PON-1 arylesterase activity is equivalent to 1 µmol of phenyl acetate hydrolyzed/min/ml.

### Statistical analysis

Statistical analyses were performed using SPSS V.19 software. Data are presented as mean and standard error (SE) for continuous variables. Comparisons between groups were done using Student’s t test for continuous variables and with the Chi square test for categorical variables. p < 0.05 was considered significant.

## Results

Out of 10 animals in the control group, one died during the two-week period before the poisoning.

**Fig. 1:**
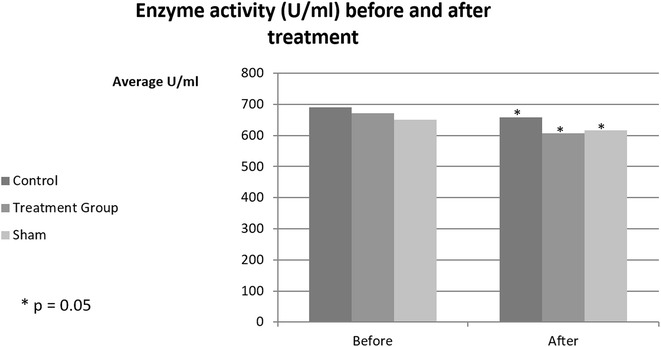
PON-1 activity levels, baseline (before exposure) and 14 days after exposure; *P = 0.05

Baseline levels of PON-1 activity were similar among the treatment, control and sham groups (p = 0.3; Table 1; Fig. 1). After 2 weeks of feeding with resveratrol, no increase in PON-1 activity level was found. On the contrary, all groups demonstrated a small yet significant decrease in the enzyme activity levels as compared to baseline levels (p = 0.05; Table 1; Fig. 1). No significant difference between the groups was demonstrated after 2 weeks, in comparison to the corresponding baseline activity levels (p = 0.7).

**Table 1:** PON-1 activity levels, before exposure (baseline, 0) and 14 days after exposure to 1.4LD50 paraoxon

	Time (days)	Average enzyme activity (U/ml)	*P*
Control group (n = 9)	0	689.8 ± 16.9	0.05
	14	657.6 ± 35.3	
Treatment group (n = 14)	0	670.5 ± 13.6	0.05
	14	607.6 ± 28.3	
Sham group (n = 6)	0	650.1 ± 20.8	0.05
	14	615.6 ± 43.2

A statistically significant and rather steady increase in body weight was observed during the 2 weeks of feeding (p < 0.01). No significant difference was noted between the study group and both the sham and control groups (p = 0.07).

All animals developed severe convulsions (score 3) and died, within 1–3 min and 10 min, respectively, following paraoxon exposure. Cause of death was respiratory failure.

## Discussion

Maintaining sufficient levels of functional cholinesterase is a major goal of a prophylactic or pre-treatment approach against NA poisoning [5]. Other requirements include also long-lasting effect, high availability, and lack of side effects. These goals could, theoretically, be achieved by various means: decreasing the levels of active OP molecules within the bloodstream, elevating the levels of cholinesterases in the bloodstream and in various tissues, elevating the concentration of endogenous and exogenous detoxifying enzymes, protecting cholinesterases from inhibition, and reactivation of inhibited enzyme [3, 5, 6].

In the current study, based on in vitro data, we have tested the efficacy of pre-treatment with resveratrol in a rat model of paraoxon poisoning. During the 14-day feeding phase preceding the exposure, we followed the animals’ weight. The change in weight implies that no serious health effects were caused by resveratrol. However, when looking at PON-1 activity levels in serum, we found no elevation in PON-1 activity levels in animals pre-treated with resveratrol. Also, all animals, including the study group, died shortly after exposure to paraoxon with no difference in survival time. Because of the rapid death, neither a comparison between the groups, nor an examination of clinical signs over an extended period of time after poisoning was possible.

There are several possible explanations for these results: (1) taking into consideration the lack of information regarding the therapeutic window of resveratrol in OP poisoning, the dose given was possibly too low to provide protection. We adjusted the dose of the compound based on the report of Williams et al. that administered resveratrol to rats at doses of 50–500 mg/kg/day during a 28-days period with no side effects [25]. The lack of a dose–response curve may lead to the theoretical possibility that the resveratrol dose was not sufficient in order to sufficiently-influence PON1 levels. However, the fact that PON1 levels did not increase and even significantly decreased during the treatment time suggest that resveratrol does not increase PON1 levels in the rat model; (2) the compound we used was of commercial origin, as a food additive, and as such contains several other compounds besides resveratrol. This may have influenced our results; (3) in this preliminary study pre-treatment was not accompanied by a standard antidotal treatment post exposure. One may argue that it should be tested in a protocol that includes post-exposure treatment. However, one of the goals in finding a new pre-treatment compound is to show it can exert its beneficial effects without post-exposure treatment. As such, resveratrol pre-treatment was found to be ineffective. In case of positive results, the next step would have been to define dose–response curves and study its role in an extended protocol including post-exposure treatment; (4) it has been reported that the bioavailability of oral resveratrol in humans is negligible, with only trace amounts of the original compound detected in the bloodstream [26]. Another study in rats showed similar results of s relatively low bioavailability [27]. Therefore, it is possible that the method of administration we used is not suitable for demonstrating the full therapeutic effect of the compound; (5) it is possible that resveratrol does not influence PON-1 levels in rats. Actually, to the best of our knowledge, this is the first time the relation between resveratrol and PON-1 is studied in vivo, in rats. (6) We did not find any data in the literature regarding PON1 levels in rats over time. We speculate that aging of the rats may have some effect on PON1 levels but this speculation is not based on hard data.

In relation to the exposure protocol, 1.4LD50 of paraoxon is a clinically significant dose, considered to be relevant for testing the efficacy and the potential role of medical countermeasures candidates, mainly in terms of counteracting severe signs of poisoning and enhancing survival, in a sort of go-no-go approach for future studies of a compound, especially in-light of the vast efforts needed to complete full development and fielding.

Another issue regards the possible role of resveratrol as post-exposure treatment. Since exposure to NAs results in a fast and severe clinical deterioration, post-exposure treatment requirements include immediate clinical effect as demonstrated, for example, by the anticholinergic drug atropine. Unfortunately, even if a compound like resveratrol has a positive effect on levels of PON1, it is notable only after a long period-of-time, thus not relevant for post-exposure treatment.

## Conclusions

Since no protection was afforded to pre-treated animals, our current results do not support the use of resveratrol as a pre-treatment measure against OP poisoning. However, this was a preliminary study, using a commercial formulation and not the pure compound. Thus, in vivo research is still relevant in order to further evaluate the possible influence of food supplements on PON-1 activity levels and resveratrol-PON1 dose–response curve in particular. Once completed, these supplements may be tested in a more advanced protocol as a protective prophylactic measure against OP poisoning.

## Abbreviations

OP: organophosphate
NA: nerve agent
PON-1: paraoxonase 1
AChE: acetylcholine esterase
BChE: butyrylcholine esterase
